# Ecosystem Consequences of Tree Monodominance for Nitrogen Cycling in Lowland Tropical Forest

**DOI:** 10.1371/journal.pone.0070491

**Published:** 2013-07-25

**Authors:** E. N. Jack Brookshire, Steven A. Thomas

**Affiliations:** 1 Department of Land Resources and Environmental Sciences, Montana State University, Bozeman, Montana, United States of America; 2 School of Natural Resources, University of Nebraska-Lincoln, Lincoln, Nebraska, United States of America; Lakehead University, Canada

## Abstract

Understanding how plant functional traits shape nutrient limitation and cycling on land is a major challenge in ecology. This is especially true for lowland forest ecosystems of the tropics which can be taxonomically and functionally diverse and rich in bioavailable nitrogen (N). In many tropical regions, however, diverse forests occur side-by-side with monodominant forest (one species >60% of canopy); the long-term biogeochemical consequences of tree monodominance are unclear. Particularly uncertain is whether the monodominant plant-soil system modifies nutrient balance at the ecosystem level. Here, we use chemical and stable isotope techniques to examine N cycling in old-growth *Mora excelsa* and diverse watershed rainforests on the island of Trinidad. Across 26 small watershed forests and 4 years, we show that *Mora* monodominance reduces bioavailable nitrate in the plant-soil system to exceedingly low levels which, in turn, results in small hydrologic and gaseous N losses at the watershed-level relative to adjacent N-rich diverse forests. Bioavailable N in soils and streams remained low and remarkably stable through time in *Mora* forests; N levels in diverse forests, on the other hand, showed high sensitivity to seasonal and inter-annual rainfall variation. Total mineral N losses from diverse forests exceeded inputs from atmospheric deposition, consistent with N saturation, while losses from *Mora* forests did not, suggesting N limitation. Our measures suggest that this difference cannot be explained by environmental factors but instead by low internal production and efficient retention of bioavailable N in the *Mora* plant-soil system. These results demonstrate ecosystem-level consequences of a tree species on the N cycle opposite to cases where trees enhance ecosystem N supply via N_2_ fixation and suggest that, over time, *Mora* monodominance may generate progressive N draw-down in the plant-soil system.

## Introduction

Tropical forests harbor an exceptional diversity of tree species and occur across a wide range of soils that vary in the availability of resources required for growth [1], [2]. Plant-soil nutrient interactions are critical to the productivity of tropical forests [3], [4], but how these interactions depend on tree diversity and associated functional traits is not straightforward. One critical problem lies in identifying the degree to which resource competition interacts with other biotic and abiotic mechanisms [1] to generate patterns in nutrient limitation and cycling at the ecosystem level. Late-successional monodominant forests (one species ≥60% of the canopy) in the tropics are a particularly vexing case as their distribution often defies simple environmental explanation [5], [6] and their persistence is, by definition, incongruous with mechanisms thought to promote high tropical tree diversity [7]–[11].

Conceptual models of monodominance have long proposed that in certain environments such as infrequently disturbed areas, combinations of species traits such as large seeds, deep litter, and shade tolerance can, over time, generate positive feedback in the plant-soil system that favors these traits [10]. Such plant functional traits express fundamental tradeoffs (e.g., rapid versus slow growth) in resource (e.g., light and nutrients) acquisition and investment. It is reasonable to expect that long-term dominance by a relatively restricted, but coordinated, set of traits would have consequences for resource availability at the ecosystem level. For example, most monodominant taxa are slow growing [9], [10] and have slow-decomposing litter [12], [13], traits associated with high plant nutrient use efficiency, low nutrient availability in soils, and small ecosystem-level nutrient losses [3]. These traits should have consequences for the nitrogen (N) cycle in particular because rates of detrital mineralization and plant N use efficiency govern N-limited plant growth late in succession [14]. Conversely, many diverse tropical forests worldwide display high levels of bioavailable N in soils and large hydrologic and gaseous N losses [15]–[18], a pattern inconsistent with N-limitation at the ecosystem level [17].

A long history of ecological research has shown that individual tree species can influence local N cycling and retention in soils and in some cases affect the balance of N inputs and losses at the ecosystem level [19], [20]. For example, trees capable of symbiotic N_2_ fixation operate to deliver new N to the ecosystem and can also increase losses of N [21], [22] but these N inputs often diminish late in forest succession [15]. In contrast, traits of many tropical monodominant species would be expected to constrain internal N turnover [9], [10] but the long-term consequences for ecosystem N losses are unknown. In theory, monodominant plant traits that act to reduce soil N to levels lower than competitors require for growth should confer a competitive advantage for N [23], [24]. If true, ecosystem losses of bioavailable N (e.g., nitrate) should be reduced relative to otherwise similar diverse forests, reflecting the strength of N limitation in each [14], [17]. We explore this possibility by asking whether the ecosystem N cycle in remote monodominant tropical forest is qualitatively and quantitatively different from adjacent diverse forests.

We examined the chemical and isotopic properties of small watershed ecosystems dominated by a well-known monodominant species, *Mora excelsa* (Bentham; *Caesalpinioideae*), and adjacent diverse watershed rainforests in Trinidad [5]. *Mora* has several traits which may confer a competitive advantage and modify soil resources: canopy dominance, shade tolerance, low-N tissues, slow litter turnover and a dense network of surface roots [25]. *Mora* does *not* form symbioses with N_2_-fixing bacteria or ectomycorrhizal fungi, but rather its roots are heavily colonized by endophytic and arbuscular mycorrhizal fungi [26]. We sampled a total of 26 small watershed forests during five spatially extensive campaigns in the Paria River basin over a four-year period. Our analyses were designed to examine if soil N properties and N losses differ between *Mora* and diverse forests and the degree to which they originate from factors internal or external to the plant-soil N cycle.

## Methods

### Ethics Statement

No permits or specific permissions were required for the location or activities of study. The study was conducted on public land and complied with all relevant regulations. Our field samples consisted of soil and water collections, which do not require permits, and did not involve endangered or protected species.

### Study Area

We studied small (5–20 ha) ungauged rainforest watersheds in the remote lower Paria River Basin (∼23 km^2^) (∼10°46′25.80″N, 61°14′57.39″W) in the Northern Range of Trinidad from 2009 to 2012. The landscape contains a dense network of small tributary watersheds and harbors the largest tract of undisturbed old-growth *Mora* rainforest in Trinidad. Mean annual precipitation (MAP) is 2750 mm distributed across distinct wet (June-December) and dry (January-May) seasons [27]. Mean annual temperature (MAT) is 25°C. Soils are Ultisols developed from low-grade meta-sedimentary phyllites of Cretaceous to Jurassic age [28], [29]. We selected small spatially-discrete watersheds in which vegetation was distinctly dominated by either *Mora* or diverse tree assemblages. *Mora* (∼90–200 meters above sea level, “MASL”) and diverse forest watersheds (∼90–300 MASL) were all upland with moderate to steep slopes (5–20%) and were not subject to flooding. All forests were old-growth with no history of logging or large natural disturbance such as fire or hurricanes [5], [30]. Diverse rainforests are classified as evergreen seasonal and lower montane forest and form a ∼30 m tall canopy composed of up to 76 tree species. In monodominant forest, *M. excelsa* accounts for >80–95% of a ∼40 m tall canopy and the majority of basal area [5], [25].

### Field and Analytical Methods

We conducted five field campaigns over three dry seasons (2009, 2010, 2011), including a severe drought (December 2009–June 2010; rainfall 43% below long-term average) and a wet dry season (2011; rainfall 46% above long-term average; Trinidad Water Resources Agency, Asa Wright Nature Centre), and two wet seasons (2011 and 2012). Our field sampling was designed to characterize N availability in the plant-soil system and N losses from watershed ecosystems dominated by *Mora* and diverse forests. Over 2009–2012 we sampled a total of 12 diverse and 14 *Mora* watersheds.

Soils were sampled in the dry seasons of 2009, 2010 and 2011 and the wet season of 2012. We sampled mineral soils (0–15 cm) from upland topographic positions by collecting cores within randomly distributed ∼5 m^2^-plots (*n = *1–3) in each of three (2009), four (2010), and eight (2011) diverse and *Mora* watersheds. Within each plot we collected and composited 3–4 individual cores. In *Mora* forests, and to a lesser extent in diverse forests, thick (∼2–15 cm) and densely rooted organic horizons form within the vicinity of individual trees [25]. In the wet season of 2012 we sampled this organic horizon and mineral soils (*n* = 3 composited cores) associated with the rooting zone (within 1 m of the trunk or buttress) of dominant canopy trees in diverse and *Mora* forests (*n = *5 plots). Field-moist, root- and rock-free soils (<2 mm) were extracted (within one day) for ammonium and nitrate in 2 M KCL. Dried soils were extracted for phosphate in weak Bray’s solution. All extracts were immediately frozen, followed by colorimetric analysis (Lachat 800 series, Zellweger Analytics) at Montana State University (MSU). Soils were also analyzed for texture hydrometrically with hexametaphosphate as a dispersant, and pH using 4∶1 water: soil solution. Dried mineral soils were analyzed for %C, %N, δ^13^C, and δ^15^N at the MBL Stable Isotope Laboratory, Woods Hole, MA.

We sampled watershed streams during the dry seasons of 2009, 2010 and 2011 and the wet season of 2011. Water samples were collected from 1^st^ and 2^nd^ order steams draining diverse and *Mora* watershed forests (*n* = 5–14) during each survey following ref 18. Though the number of watersheds sampled varied across years, a minimum of 5 watersheds of each forest type were sampled every year. Rainfall was collected during 22 rain events over 2008–2011 in an open area at 330 MASL within ∼5 km from the study sites using clean and leached funnel collectors designed to capture bulk inorganic N deposition [19]. We consider this location to represent an upper-end estimate of bulk wet inorganic N deposition to the study area as the collection site is slightly higher in elevation and receives higher orographically-driven rainfall (>3000 mm). All rain and stream water samples were field-filtered (0.7 mm glass fiber) into triple-leached polyurethane bottles and frozen until analysis [19]. Water samples were analyzed for anions via flow injection spectrophotometry (SEAL QuAATro, Mequon WI) and ion chromatography (Metrohm, Riverview FL), cations via ion chromatography and inductively coupled plasma optical emission spectrometry (Optimum 5300 DV, Perkins Elmer), δ^2^H and δ^18^O via laser absorption liquid-water isotope spectrometry (Model DLT-100, Los Gatos Research), and DOC and total dissolved N (TDN) via high temperature platinum combustion with a total N module (Shimadzu TOC-V) at MSU, and δ^15^NO_3_ via the denitrifier method and isotope ratio mass spectrometry (UC Davis Stable Isotope Facility, Davis, CA). Deuterium-excess was calculated relative to the global meteoric water line: δ^2^H – (8 *δ^18^O) Dissolved organic N (DON) was calculated as TDN – (nitrate+ammonium).

We estimated atmospheric N deposition fluxes as the geometric mean and associated quadratic error of the product of DIN (nitrate+ammonium) concentrations in rain for each event and MAP. As all of our study watersheds are ungauged, we estimated hydrologic N losses using seasonal rainfall levels for each sampling period over 2009–2011 by applying a 50% water loss via evapotranspiration (ET, based on regional estimates for sites with similar MAP [31]) and multiplying resulting runoff by individual stream N concentrations. This approach assumes similar ET characteristics between forest types, such as rates of canopy interception and evaporation as we currently lack data on such processes. However, we evaluated this assumption by analyzing stream samples for chemical and isotopic properties (e.g., Cl^−^ and deuterium excess) that are sensitive to watershed-level effects of dilution and evaporation. We calculated annual fluxes and associated error via quadratic error propagation using observed seasonal variation in calculated stream discharge and stream N concentrations. We estimated gas losses via denitrification using a simple steady-state isotope mass balance model [16]. We parameterized the model using observed mean bulk soil ^15^N, mean atmospheric deposition ^15^N, an isotope effect for leaching calculated by subtracting observed mean ^15^NO_3_ in streams from mean bulk soil ^15^N for each forest type, and a 16‰ isotope enrichment effect for denitrification following ref 16. We estimated error in total N losses via quadratic error propagation assuming that variance in fractional gas losses varied proportionally with the observed standard deviation in ^15^NO_3_ and combining this with the observed standard deviation in hydrologic N fluxes across all samples.

Differences in bulk soil physical, chemical and isotopic properties between forests types were tested using t-tests. Differences in levels of bioavailable soil N between forests and over time were examined using two-way ANOVA on log-transformed values followed by Tukey *post hoc* tests. For years in which multiple plots were sampled within watersheds (2009, 2010) initial examination (ANOVA) indicated a non-significant effect of watershed identity on soil chemistry. We therefore treat these plots as independent samples (2009, *n* = 6; 2010, *n* = 12; 2011, *n* = 8) in tests of forest-type effects. Stream chemical and isotopic data were analyzed using a combination of linear regression and ANOVA (all statistical tests were made using SigmaPlot). Initial analyses (ANOVA) revealed that watershed identity had no effect on intra- and inter-forest temporal and spatial patterns in stream nitrate and therefore individual watershed-years were treated as independent samples in subsequent tests.

## Results and Discussion

We first examined bulk soil physical and chemical properties (bulk density, texture, pH, %C, %N or δ^13^C, and δ^15^N) that integrate long-term organic matter processing in the plant-soil system [32]. We found no differences in bulk density, texture, %C, %N or δ^13^C in shallow (0–15 cm) mineral soils of diverse versus *Mora* forests and only marginal (*P = *0.06; two-tailed t test) differences in pH (Table 1) implying similar long-term organic matter storage across these forests. We also found no differences between forests in measures of extractable soil phosphate. Despite equivalent levels of bulk soil N, however, we found marginally (*P* = 0.06; two-tailed t test) higher levels of δ^15^N in soils of diverse compared to *Mora* forests a pattern that implies higher N availability and microbial cycling [18] within the plant-soil N system of diverse forests.

**Table 1:** Physical, chemical, isotopic and biological properties of bulk mineral soils (0–15 cm).

Soil Property	*Mora*	Diverse	*p-value*
Bulk density (g/cm^3^)	1.3 (0.1)	1.2 (0.2)	*0.32*
Clay (%)	36.2 (1.8)	31.5 (3.5)	*0.26*
Silt (%)	38.2 (1.7)	36.2 (3.1)	*0.59*
Sand (%)	25.5 (2.7)	32.3 (6.4)	*0.36*
pH	3.9 (3.7–4.1)	4.1 (3.7–4.5)	*0.06*
C (%)	3.1 (0.5)	2.6 (0.4)	*0.27*
N (%)	0.3 (0.02)	0.3 (0.03)	*0.47*
δ^13^C (‰)	−28.9 (0.1)	−28.8 (0.3)	*0.40*
δ^15^N (‰)	4.7 (0.3)	5.7 (0.4)	*0.06*
Bray’s-P (µg/g)	2.6 (0.4)	2.9 (0.5)	*0.52*
Root biomass (g/m^2^)	129.4 (45.2)	70.0 (48.8)	*0.39*

Values are means and 1 SE (in parentheses) for watershed-level composited samples for bulk density (*n* = 10); texture (*n* = 5); pH (*n* = 15); %C, %N, δ^13^C and δ^15^N (*n* = 8); and Brays-P (*n* = 15); and root biomass (*n* = 5), where the sample size (*n*) refers to the number of plots. For pH, the mean was calculated by averaging hydrogen ion concentrations followed by log10 back transformation. The values in parentheses are quadratic ranges. P-values are the results of two-sided t-tests.

**Figure 1:**
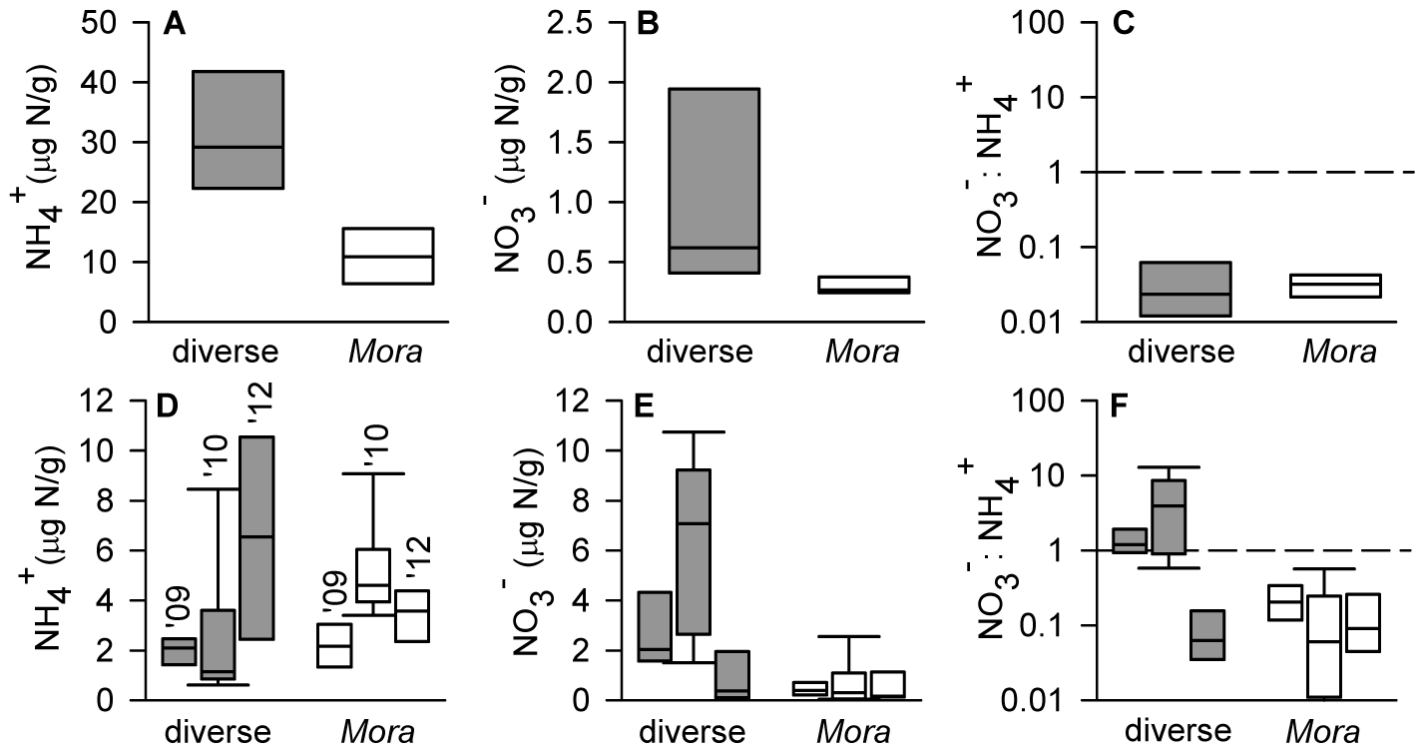
Soil N availability in monodominant (*Mora*) and diverse rain forests in Trinidad. (A–C) Box plots (median, 5^th^ and 95^th^ percentiles) of ammonium, nitrate, and nitrate: ammonium ratios in surface organic soils near focal trees collected during the wet season (2012). Levels of ammonium (*P* = 0.007, two-tailed t test) and nitrate (*P* = 0.073, two-tailed t test) were lower in soils of *Mora* forests (white symbols) than in diverse forests (grey symbols) but nitrate: ammonium ratios did not differ (*P* = 0.85, two-tailed t test). (D, E) Box plots (median, 5^th^ and 95^th^ percentiles) showing levels of ammonium and nitrate in mineral (0–15 cm) soils during dry (2009 and 2010) and wet (2012) seasons. Soils were not measured for inorganic N in 2011. Across years ammonium varied significantly (*P* = 0.017 for year effect) but did not differ consistently between forest types (*P* = 0.26 for type effect) but the effect of year depended on forest type (*P*<0.001 for interaction term, two-way ANOVA). In contrast, nitrate was consistently lower in *Mora* than diverse forests across years and did not change through time whereas nitrate in diverse forests showed increases during dry seasons (*P*<0.001 for type effect, *P* = 0.029 for year effect, and *P* = 0.009 for interaction term, two-way ANOVA). This resulted in low and stable nitrate: ammonium ratios in *Mora* compared to diverse forests (*P*<0.001 for all effects, two-way ANOVA).

Analysis of plant available N (ammonium and nitrate) in soil extracts supported this inference. Our sampling across wet and dry seasons revealed that concentrations of mineral N were consistently lower in soils of *Mora* compared to diverse forests (Fig. 1A, 1B, 1D, 1E). During the wet season, ammonium was >3 times lower and nitrate >2 times lower within the organic rooting zone of individual *Mora* trees than under dominant taxa in diverse forests (Fig. 1A, 1B), consistent with low N content and turnover [25] of *Mora* litter. Further, root biomass in this organic layer was twice as high in *Mora* (average ± SEM, 333±75 g/m^2^) than diverse (average ± SEM, 166±51 g/m^2^) forests (*P = *0.056****Mann-Whitney Rank Sum Test). These differences in the organic layer would also predict low nitrate accumulation in deeper soils. Accordingly, we found that mineral soils of diverse forests contained much higher nitrate levels, independent of season, that were characteristic of N-rich ecosystems [18], while nitrate in *Mora* forests was consistently low (Fig. 1B, 1E). Ammonium was always the dominant form of mineral N in *Mora* soils, a pattern common to N-limited temperate forests [33]. In contrast, high nitrate: ammonium ratios generally occur under conditions of high N availability where nitrate can accumulate [34]. During the wet season, ammonium was the dominant form of available N in all soils and resulted in low nitrate: ammonium ratios, particularly in organic horizons (Fig. 1C). Across all dry season samples, however, nitrate: ammonium ratios were >10–100 times higher in diverse compared to *Mora* forests (Fig. 1F). Concentrations of inorganic N in *Mora* forests remained unchanged across wet and dry seasons over a three year period. In contrast, over the same seasonal sequence diverse forests showed a significant decrease in ammonium, a six-fold increase in nitrate, and a fifty-fold increase in nitrate: ammonium ratios.

This difference could not be explained by differences in soil moisture (Fig. S1) suggesting that low bioavailable N in *Mora* forests does not result from differential availability of water between forest types but, rather, differential sensitivity of the N cycle to rainfall. Low and unvarying levels of bioavailable N indicate remarkable stability of the internal *Mora* N cycle despite large seasonal variation in rainfall. Large seasonal variation in diverse forests on the other hand is consistent with an internal N cycle sensitive to moisture and high microbial nitrate production [35] leading to high mineral N losses from the plant-soil ecosystem [18].

**Figure 2:**
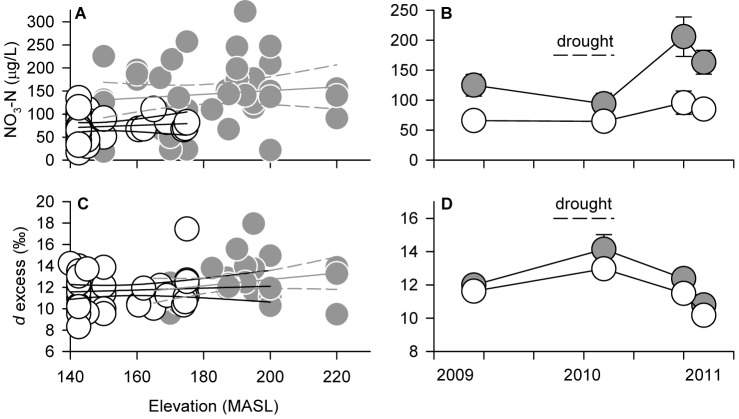
Spatial and temporal distribution of nitrate and *d*-excess in small watershed streams. (A) Across all samples stream nitrate did not change as a function of watershed elevation (median of ridge top and watershed outlet elevations) within *Mora* (white symbols, *n* = 38) or diverse (grey symbols, *n* = 40) watersheds or across all watersheds combined (*P*>0.2 for all comparisons, linear regression and 95% CI). (*B*) Mean (± SEM) stream nitrate concentrations over the 2009–2011 period in *Mora* (*n* = 5, 13, 6, and 14) and diverse (*n* = 12, 9, 6, and 12) watersheds. Nitrate in stream waters was consistently higher and more variable in diverse than *Mora* forests across years (*P*<0.001 for type effect, *P*<0.001 for year effect, and *P* = 0.028 for interaction term, two-way ANOVA). Across the drought to post-drought sequence nitrate in *Mora* forest remained unchanged (*P*>0.57) while nitrate in diverse forests increased significantly (*P<*0.01). (C) Across all samples *d*-excess did not change as a function of elevation within *Mora* (white symbols, *n* = 32) or diverse (grey symbols, *n* = 30) watersheds or across all watersheds combined (*P*>0.16 for all comparisons, linear regression and 95% CI). (D) Mean (± SEM) *d*-excess in stream waters varied significantly and synchronously across time (*P*<0.001 for year effect) but did not differ between forest types across or within years (*P* = 0.104 for type effect, *P* = 0.933 for interaction term, two-way ANOVA).

We tested this prediction by analyzing dissolved N in small watershed streams. Across all forests and sampling periods, our analysis revealed ∼2-fold lower concentrations of dissolved inorganic N (DIN) in streams draining *Mora* forests than in diverse forests. Nitrate was significantly lower (*P*<0.001, two-way ANOVA) in *Mora* forests compared to its diverse neighbors (Fig. 2A, 2B) while concentrations of ammonium were low across all forests (average ± SEM, 5±0.4 µg N/L, *n* = 78) and did not differ between forest types. Stream nitrate concentrations in our diverse forests are similar to those reported for other N-rich tropical forests while those in *Mora* forests are at the low end of observations for forests of similar climate worldwide [15], [17].

Several physical factors external to the plant-soil system could give rise to lower nitrate concentrations in streams draining *Mora* forests. For example, the propensity for N losses could change systematically with elevation or slope. Diverse forests occupy a broader topographic range than *Mora*, but we observed constant between-forest differences and within-forest variance in nitrate across all elevations (Fig. 2A). We also considered whether differences in watershed hydrologic fluxes could explain the nitrate pattern. Diluted nitrate concentrations could result from higher runoff or lower rates of evaporation in *Mora* forests. In the former case we would predict proportional dilution of more biologically conservative solutes such as Cl^−^, SO4^2−^, or base cations. However, we found no differences in concentrations of cations or SO4^2−^ between forest types, suggesting similar runoff characteristics among watersheds, and only marginally higher levels of Cl^−^ in *Mora* forests (Fig. S2), a pattern opposite to that predicted if dilution explained the nitrate pattern. Similarly, we evaluated whether evaporation differences among watersheds might control nitrate concentrations by examining distributions of stable water isotopes in stream water. Across all samples, deuterium excess, which traces local water sources and evaporation [36], did not change with elevation nor differ between forest types over time (Fig. 2C, 2D). We conclude that differences in topography, runoff, and evaporation are unlikely to explain the inter-forest differences in nitrate losses observed in these catchments.

We next examined the sensitivity of stream nitrate to inter-annual variation in rainfall. Figure 2B illustrates that nitrate remained lower in *Mora* than diverse forests prior to, over the course of, and following a severe drought (Dec. 2009–Jun. 2010). Over the drought-to-wet season sequence, stream nitrate in diverse forests showed a dramatic and sustained two-fold increase, while nitrate in *Mora* forests remained unchanged. Large increases in nitrate concentration with increasing discharge are typically observed in ecosystems with high internal nitrate production and nitrate accumulation in soils [18]. Consistently higher nitrate in soils of diverse than *Mora* forests corroborates that this difference is an internally generated and temporally stable feature of the N cycle of these ecosystems, at least over the period of our study.

Using N concentrations from all streams and basin-scale water fluxes we estimate that exports of DIN are ∼2.6 kg N ha yr^−1^ (2.2–3.1 kg N ha yr^−1^, quadratic range) from diverse forests and ∼1.1 kg N ha yr^−1^ (1.0–1.3 kg N ha yr^−1^) from *Mora* forests. To sustain such differences in hydrologic N losses from these old-growth forests requires differences in other pathways of N loss or differences in external inputs of N [14]. As these forests are all old-growth and have acid soils, N losses from fire or volatilization are unlikely vectors. Instead, we focus on losses via dissolved organic N (DON) leaching and gaseous N via denitrification since the former has been shown to sustain N limitation in old forests [14] and the latter can be significant in N-rich tropical forests [16].

**Figure 3:**
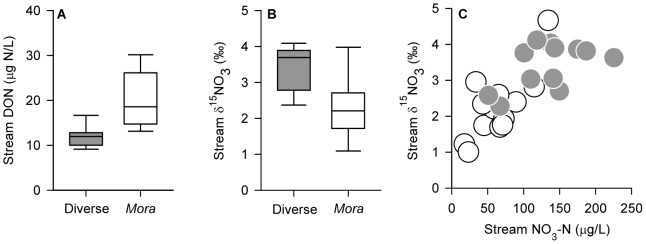
Organic nitrogen and natural abundance isotope distributions of nitrate in streams. (A) Box plots (median, 5^th^ and 95^th^ percentiles) of dissolved organic N (DON) concentrations in streams of diverse (*n* = 9) and *Mora* (*n* = 15) forests. DON was significantly higher (*P*<0.001, two-tailed t test) in *Mora* than diverse forests. (B) Box plots (median, 5^th^ and 95^th^ percentiles) showing significantly higher (*P* = 0.001, two-tailed t test) δ^15^NO_3_ in streams of diverse (*n* = 12) than *Mora* (*n = *13) forests. (C) δ^15^NO_3_ in streams increased as a function of stream nitrate concentrations (r^2^ = 0.57, *P*<0.001, linear regression) across all forests.

Across all our study watersheds we found low levels of stream dissolved organic carbon (DOC, 0.1–1.1 mg/L) and DON but that levels of DON were on average higher (*P*<0.01; two-tailed t test) in streams draining *Mora* (Fig. 3A). However, this difference could not balance the difference in dissolved N losses between forests (accounting for <5–20% of total dissolved N losses). Alternatively, elevated gaseous losses via denitrification could explain the observed imbalance. We examined this possibility by first analyzing the natural abundance δ^15^N distributions of stream water nitrate. We observed 1) δ^15^NO_3_ was significantly higher in diverse forest streams than *Mora* streams (Fig. 3B), and 2) δ^15^NO_3_ became increasingly enriched with increasing nitrate concentrations (Fig. 3C), a pattern expected from microbial denitrification [18] and consistent with enriched soil ^15^N in diverse forests soils. Combined, these results suggest an ecosystem-level signature of denitrification opposite that expected if denitrification accounted for the observed N imbalance, supporting our inference that total mineral N losses are greater from diverse forests compared to *Mora* forests.

We next examined the contribution of gaseous losses to total N losses from these forests and how these compare to abiotic N inputs via atmospheric deposition. We achieved this in two ways: 1) direct measures of DIN and δ^15^NO_3_^−^ in rain water; and 2) N isotope mass balance. We combined DIN concentrations from 22 rain events sampled over four years with mean annual precipitation to estimate bulk inorganic N deposition inputs (2.7 kg N ha^−1^ yr^−1^; quadratic range: 1.3–5.6 kg N ha^−1^ yr^−1^) consistent with previous field [18] and model [37] estimates of reactive N deposition in the region. We estimated N gas losses using an isotope mass balance model [16] parameterized with direct measures of δ^15^N in bulk soils (Table 1), δ^15^NO_3_^−^ in stream water (Fig. 3B and 3C) and precipitation (average ± SEM, 1.4±0.7‰, *n = *11), and a mean fractionation factor for soils from the literature [16]. This approach yielded denitrification rates equal to 49% and 44% of total N losses from diverse and *Mora* forests, respectively. Combining gaseous losses with hydrologic N fluxes, we estimate that total inorganic N losses are ∼5.1 kg N ha^−1^ yr^−1^ (quadratic range: 4.3–5.9 kg N ha^−1^ yr^−1^) from diverse forests and ∼2.0 kg N ha^−1^ yr^−1^ (quadratic range: 1.7–2.3 kg N ha^−1^ yr^−1^) from *Mora* forests, resulting in inorganic N loss: input ratios >1 in diverse forests and <1 in *Mora* forests. Importantly, we find that leaching losses approximately balance inputs in diverse but not *Mora* forests even if we exclude gaseous losses entirely. We conclude that bioavailable N losses likely exceed abiotic inputs in diverse forests but are unlikely to in *Mora* forests (Table 2). This implies a fundamental difference in the N cycle of these forests, as the former pattern suggests the lack of N limitation, while the latter suggests its possibility [14].

**Table 2:** Ecosystem mineral N balance for diverse and monodominant forests (kg N ha^−1^ yr^−1^).

	Ecosystem losses		
	NO_3_^−^+NH_4_^+^	N gas	*Loss: Inputs*
*Mora*	1.1 (1.0–1.3)	0.9 (0.7–1.0)	0.7
diverse	2.6 (2.2–3.1)	2.4 (2.1–2.8)	1.9

Hydrologic losses were estimated as the geometric mean of the product of stream DIN concentration by stream water efflux (assuming evapotranspiration of 50%). Gas losses were calculated using a simple isotope mass balance approach [16] parameterized with measured isotope values in rain water, soils, and streams. Values in parentheses are quadratic ranges (error) of fluxes. Mineral N loss: input ratios are calculated as hydrologic+gaseous losses divided by atmospheric deposition fluxes (2.7 kg ha^−1^ yr^−1^).

For these differences in N losses to be sustained, as our evidence suggests, requires that external inputs of N to diverse forests exceed those to *Mora* forests. That is, assuming that soil N is near steady state our finding of no differences in bulk soil %N (Table 1) but large and sustained differences in N losses between forests means that N inputs likely differ. This could result from higher rates of biological N_2_ fixation associated with diverse forests [15]. In addition to widespread asymbiotic N_2_ fixation in tropical forests in general [38], our study area harbors leguminous tree species (e.g., *Pentaclethra macroloba*, *Inga* sp.) known to fix large amounts of N [15], [39], but which are less abundant in *Mora* forest [5], [25]. Given its extreme canopy, understory and litter-layer dominance, it is possible that *Mora* acts to exclude these potential N fixers despite lower soil N availability. In contrast to symbiotic N_2_ fixation, however, the C-rich and low inorganic N root-litter layer of *Mora* forests would seem to favor asymbiotic N_2_ fixers [15] but we currently lack data on this process. At the same time, if this external input is significant in *Mora* forests, our results suggest that biological cycling of this new N must be extremely efficient and/or or that total N pools are still growing or that higher DON losses balance this input given that mineral N pools and losses are small. Lower N supply should in turn favor plant traits which tightly conserve N in the plant-soil system.

*Mora* has low foliar and litter N concentrations and low litterfall N fluxes [25] relative to other tropical trees globally [40], [41] and shows significant foliar resorption of N [25]. Slow decomposition rates cause *Mora* litter to accumulate and it has been speculated that it is from this soil layer that surface roots of *Mora* procure their nutrient requirements [25]. Nitrate levels in *Mora* soils are exceptionally low and similar to those found in N-limited temperate forests [33]. Low soil nitrate has also been found in the monodominant *Gilbertiodendron dewevrei* (*Caesalpinioideae*) forests of Africa [9]. Such low nitrate relative to other tropical forests could result from preferential plant drawdown of ammonium (by reducing the ammonium pool available to microbial nitrifiers) or nitrate [42] and/or a variety of microbial processes that act to lower nitrate production [43]–[45]. These mechanisms are consistent with the theoretical prediction that relatively high plant preference for ammonium and ability to inhibit nitrification can allow for invasion of and resistance to invasion by, plants without these traits [42]. These mechanisms could be examined via a combination of reciprocal litter and seedling transplant experiments and N fertilization and ^15^N-tracer additions. Irrespective of the plant-microbial mechanism/s underlying low soil nitrate, however, we show that low soil nitrate associated with monodominance has ecosystem-level consequences for patterns of N loss. This results from the fact that nitrate is both highly mobile in soils and is also the substrate for microbial denitrification. Thus lower nitrate in soils should translate directly to smaller hydrologic and gaseous N losses at the ecosystem level [15], [18], which is what we observed.

Though it is reasonable that known functional traits of *Mora* (canopy dominance, shade tolerance, deep litter) would provide a competitive advantage in some environments, it remains unknown how *Mora* comes to dominate otherwise diverse landscapes and the degree to which this depends on initial conditions, including characteristics of the physical environment, and disturbance and invasion dynamics. Here, we find that *Mora* dominance late in forest succession is associated with strong reductions in levels of bioavailable N in the plant-soil system and losses at the ecosystem level. This differentiates *Mora* from other trait-driven N cycles in which tree species increase ecosystem N supply through symbiotic N_2_ fixation following disturbance or invasion [21], [22]. Curiously, the majority of monodominant taxa worldwide are *Caesalpinioideae* legumes [10], a group in which symbiotic N_2_-fixation is rare [46]. In many diverse tropical forests, N_2_ fixation by legumes may drive post-disturbance N accumulation [34] but this input slows via negative plant-soil feedback as available N pools increase [15], [39]. Positive or self-reinforcing feedback, on the other hand, tends to destabilize plant-nutrient systems [47]. For the *Mora* system, if positive N feedback is operating, progressive draw-down of available N could ultimately exacerbate N limitation and could both drive selection toward increasing N use efficiency but also increase system sensitivity to perturbations that alter the competitive environment. We suggest that forest monodominance in the tropics provides a promising model system for exploring the organization of the tropical N cycle in particular and higher-order consequences of species functional traits in general.

## Supporting Information

Figure S1 **Concentrations of soil ammonium and nitrate as a function of gravimetric soil moisture.** A, B) Concentrations of ammonium and nitrate in organic horizons of forest soils during the wet season (2012). C, D) Concentrations of ammonium and nitrate in mineral (0–15 cm) soils during dry (2010; triangles) and wet (2012; circles) seasons. Variation in soil moisture did not explain any significant (*P*>0.20) within- or between-forest variation in soil N.
(TIF)

Figure S2 **Concentrations of anions and cations in small watershed streams of diverse and** ***Mora*** **forests of Trinidad.** (A) Box plots (median, 5^th^ and 95^th^ percentiles and outliers) of stream chloride in diverse (grey symbols *n* = 18) and *Mora* (white symbols, *n* = 15) forests. Stream chloride decreased strongly and synchronously between dry (2010) and wet (2011) years (*P*<0.001 for year effect) and differed between forest types (*P* = 0. 013 for type effect) with no significant year-type interaction (*P* = 0.139 for interaction term, df = 26, two-way ANOVA). Slightly higher chloride in *Mora* forest is opposite to that expected if dilution explained lower stream nitrate, is not consistent with other ions and water isotopes and could be explained by slightly closer proximity to the ocean. (B) Box plots (median, 5^th^ and 95^th^ percentiles) of stream sulphate in diverse (*n* = 18) and *Mora* (*n* = 15) forests. Sulphate also decreased significantly and synchronously from dry to wet seasons (*P*<0.001 for year effect) but did not differ between forest types across or within years (*P* = 0.098 for type effect, *P* = 0.611 for interaction term, df = 26, two-way ANOVA). (C) Box plots (median, 5^th^ and 95^th^ percentiles) of the sum of stream cations (K^+^, Na^+^, Ca^++^, Mg^++^). Cations showed significant and synchronous increases from 2009 to 2010 (*P*<0.001 for year effect) and no differences between forest types across or within years (*P* = 0.227 for type effect, *P* = 0.116 for interaction term, df = 29, two-way ANOVA).
(TIF)

## References

[1] Wright SJ (2002) Plant diversity in tropical forests: a review of mechanisms of species coexistence. Oecologia 130: 1–14. 28547014 10.1007/s004420100809

[2] Wright SJ, Yavitt JB, Wurzburger N, Turner BL, Tanner EVJ, et al (2011) Potassium, phosphorus, or nitrogen limit root allocation, tree growth, or litter production in a lowland tropical forest. Ecology 92: 1616–1625. 21905428 10.1890/10-1558.1

[3] Vitousek P (2004) Nutrient cycling and limitation, Hawai’i as a model system. Princeton University Press.

[4] Townsend AR, Cleveland CC, Houlton BZ, Alden CB, White JWC (2011) Multi-element regulation of the tropical forest carbon cycle. Front Ecol Environ 9: 9–17.

[5] Beard J (1946) The Mora forests of Trinidad, British West-Indies. Journal of Ecology 33: 173–192.

[6] Peh KS-H, Sonke B, Lloyd J, Quesada CA, Lewis SL (2011) Soil does not explain monodominance in a central African tropical forest. PLoS One 6. doi:10.1371/journal.pone.0016996 10.1371/journal.pone.0016996 PMC3037391 21347320

[7] Connell J, Lowman M (1989) Low-diversity tropical rain forests - some possible mechanisms for their existence. American Naturalist 134: 88–119.

[8] Hart T (1990) Monospecific dominance in tropical rain forests. Trends in Ecology & Evolution 5: 6–11. 21232309 10.1016/0169-5347(90)90005-X

[9] Torti S, Coley P, Kursar T (2001) Causes and consequences of monodominance in tropical lowland forests. American Naturalist 157: 141–153. 10.1086/318629 18707268

[10] Peh KS-H, Lewis SL, Lloyd J (2011) Mechanisms of monodominance in diverse tropical tree-dominated systems. J Ecol 99: 891–898.

[11] Mangan SA, Schnitzer SA, Herre EA, Mack KML, Valencia MC, et al (2010) Negative plant-soil feedback predicts tree-species relative abundance in a tropical forest. Nature 466: 752–755. 20581819 10.1038/nature09273

[12] McGuire KL, Zak DR, Edwards IP, Blackwood CB, Upchurch R (2010) Slowed decomposition is biotically mediated in an ectomycorrhizal, tropical rain forest. Oecologia 164: 785–795. 20577764 10.1007/s00442-010-1686-1

[13] Peh KS-H, Sonke B, Taedoung H, Sene O, Lloyd J, et al (2012) Investigating diversity dependence of tropical forest litter decomposition: experiments and observations from Central Africa. J Veg Sci 23: 223–235.

[14] Menge DNL (2011) Conditions under which nitrogen can limit steady-state net primary production in a general class of ecosystem models. Ecosystems 14: 519–532.

[15] Hedin L, Brookshire E, Menge D, Barron A (2009) The nitrogen paradox in tropical forest ecosystems. Annual Review of Ecology Evolution and Systematics 40: 613–635.

[16] Houlton B, Bai E (2009) Imprint of denitrifying bacteria on the global terrestrial biosphere. Proceedings of the National Academy of Sciences of the United States of America 106: 21713–21716. 19995974 10.1073/pnas.0912111106 PMC2789759

[17] Brookshire ENJ, Gerber S, Menge DNL, Hedin LO (2012) Large losses of inorganic nitrogen from tropical rainforests suggest a lack of nitrogen limitation. Ecol Lett 15: 9–16. 22017659 10.1111/j.1461-0248.2011.01701.x

[18] Brookshire ENJ, Hedin LO, Newbold JD, Sigman DM, Jackson JK (2012) Sustained losses of bioavailable nitrogen from montane tropical forests. Nat Geosci 5: 123–126.

[19] Zinke PJ (1962) The pattern of influence of individual forest trees on soil properties. Ecology 43: 130–133.

[20] Binkley D, Giardina C (1998) Why do tree species affect soils? The warp and woof of tree-soil interactions. Biogeochemistry 42: 89–106.

[21] Vitousek PM, Walker LR (1989) Biological Invasion by Myrica faya in Hawai’i: Plant Demography, Nitrogen Fixation, Ecosystem Effects. Ecological Monographs 59: 247–265.

[22] Compton JE, Church MR, Larned ST, Hogsett WE (2003) Nitrogen export from forested watersheds in the Oregon Coast Range: The role of N(2)-fixing red alder. Ecosystems 6: 773–785.

[23] Tilman D (1980) Resources - a Graphical-Mechanistic Approach to Competition and Predation. Am Nat 116: 362–393.

[24] Daufresne T, Hedin LO (2005) Plant coexistence depends on ecosystem nutrient cycles: extension of the resource-ratio theory. Proceedings of the National Academy of Sciences of the United States of America 102: 9212–9217. 15964989 10.1073/pnas.0406427102 PMC1166585

[25] Cornforth IS (1970) Leaf-fall in a tropical rain forest. Journal of Applied Ecology 7: 603–608.

[26] Torti S, Coley P, Janos D (1997) Vesicular-arbuscular mycorrhizae in two tropical monodominant trees. Journal of Tropical Ecology 13: 623–629.

[27] Shrivastava GS (2003) Estimation of sustainable yield of some rivers in Trinidad. J Hydrol Eng 8: 35–40.

[28] Frey M, Saunders J, Schwander H (1988) The Mineralogy and Metamorphic Geology of Low-Grade Metasediments, Northern Range, Trinidad. J Geol Soc 145: 563–575.

[29] Ahmad N, Jones R, Beavers A (1968) Genesis mineralogy and related properties of West Indian Soils.2. maracas series formed from micaceous schist and phyllite Northern Range Trinidad. Journal of Soil Science 19: 9–19.

[30] Helmer EH, Ruzycki TS, Benner J, Voggesser SM, Scobie BP, et al (2012) Detailed maps of tropical forest types are within reach: Forest tree communities for Trinidad and Tobago mapped with multiseason Landsat and multiseason fine-resolution imagery. For Ecol Manage 279: 147–166.

[31] Rollenbeck R, Anhuf D (2007) Characteristics of the water and energy balance in an Amazonian lowland rainforest in Venezuela and the impact of the ENSO-cycle. J Hydrol 337: 377–390.

[32] Baisden WT, Amundson R, Cook AC, Brenner DL (2002) Turnover and storage of C and N in five density fractions from California annual grassland surface soils. Glob Biogeochem Cycle 16. doi:10.1029/2001GB001822

[33] Perakis SS, Hedin LO (2001) Fluxes and fates of nitrogen in soil of an unpolluted old-growth temperate forest, southern Chile. Ecology 82: 2245–2260.

[34] Davidson E, de Carvalho C, Figueira A, Ishida F, Ometto J, et al (2007) Recuperation of nitrogen cycling in Amazonian forests following agricultural abandonment. Nature 447: 995–U6. 17581583 10.1038/nature05900

[35] Davidson E, Nepstad D, Ishida F, Brando P (2008) Effects of an experimental drought and recovery on soil emissions of carbon dioxide, methane, nitrous oxide, and nitric oxide in a moist tropical forest. Global Change Biology 14: 2582–2590.

[36] Brooks J, Barnard H, Coulombe R, McDonnell J (2010) Ecohydrologic separation of water between trees and streams in a Mediterranean climate. Nature Geoscience 3: 100–104.

[37] Dentener F, Drevet J, Lamarque J, Bey I, Eickhout B, et al. (2006) Nitrogen and sulfur deposition on regional and global scales: A multimodel evaluation. Global Biogeochemical Cycles 20. doi:10.1029/2005GB002672

[38] Reed SC, Cleveland CC, Townsend AR (2011) Functional ecology of free-living nitrogen fixation: a contemporary perspective. Annual Review of Ecology, Evolution, and Systematics, Vol. 42: 489–512.

[39] Barron AR, Purves DW, Hedin LO (2010) Facultative nitrogen fixation by canopy legumes in a lowland tropical forest. Oecologia 165: 511–520. 21110206 10.1007/s00442-010-1838-3

[40] Vitousek PM (1984) Litterfall, nutrient cycling, and nutrient limitation in tropical forests. Ecology 65: 285–298.

[41] Townsend A, Cleveland C, Asner G, Bustamante M (2007) Controls over foliar N: P ratios in tropical rain forests. Ecology 88: 107–118. 17489459 10.1890/0012-9658(2007)88[107:cofnri]2.0.co;2

[42] Boudsocq S, Niboyet A, Lata JC, Raynaud X, Loeuille N, et al (2012) Plant preference for ammonium versus nitrate: a neglected determinant of ecosystem functioning? Am Nat 180: 60–69. 22673651 10.1086/665997

[43] Subbarao GV, Rondon M, Ito O, Ishikawa T, Rao IM, et al (2007) Biological nitrification inhibition (BNI) - is it a widespread phenomenon? Plant Soil 294: 5–18.

[44] Silver W, Herman D, Firestone M (2001) Dissimilatory nitrate reduction to ammonium in upland tropical forest soils. Ecology 82: 2410–2416.

[45] Wieder WR, Cleveland CC, Taylor PG, Nemergut DR, Hinckley E-L, et al (2013) Experimental removal and addition of leaf litter inputs reduces nitrate production and loss in a lowland tropical forest. Biogeochemistry 113: 629–642.

[46] Sprent JI, James EK (2007) Legume evolution: Where do nodules and mycorrhizas fit in? Plant Physiol 144: 575–581. 17556520 10.1104/pp.107.096156 PMC1914177

[47] Deangelis D, Mulholland P, Palumbo A, Steinman A, Huston M, et al (1989) Nutrient dynamics and food-web stability. Annual Review of Ecology, Evolution, and Systematics 20: 71–95.

