# Supplementary figures and images for: Ecosystem Consequences of Tree Monodominance for Nitrogen Cycling in Lowland Tropical Forest
Source: PLoS One. 2013 Jul 25;8(7):e70491. doi: 10.1371/journal.pone.0070491 (PMC3723728; doi:10.1371/journal.pone.0070491)

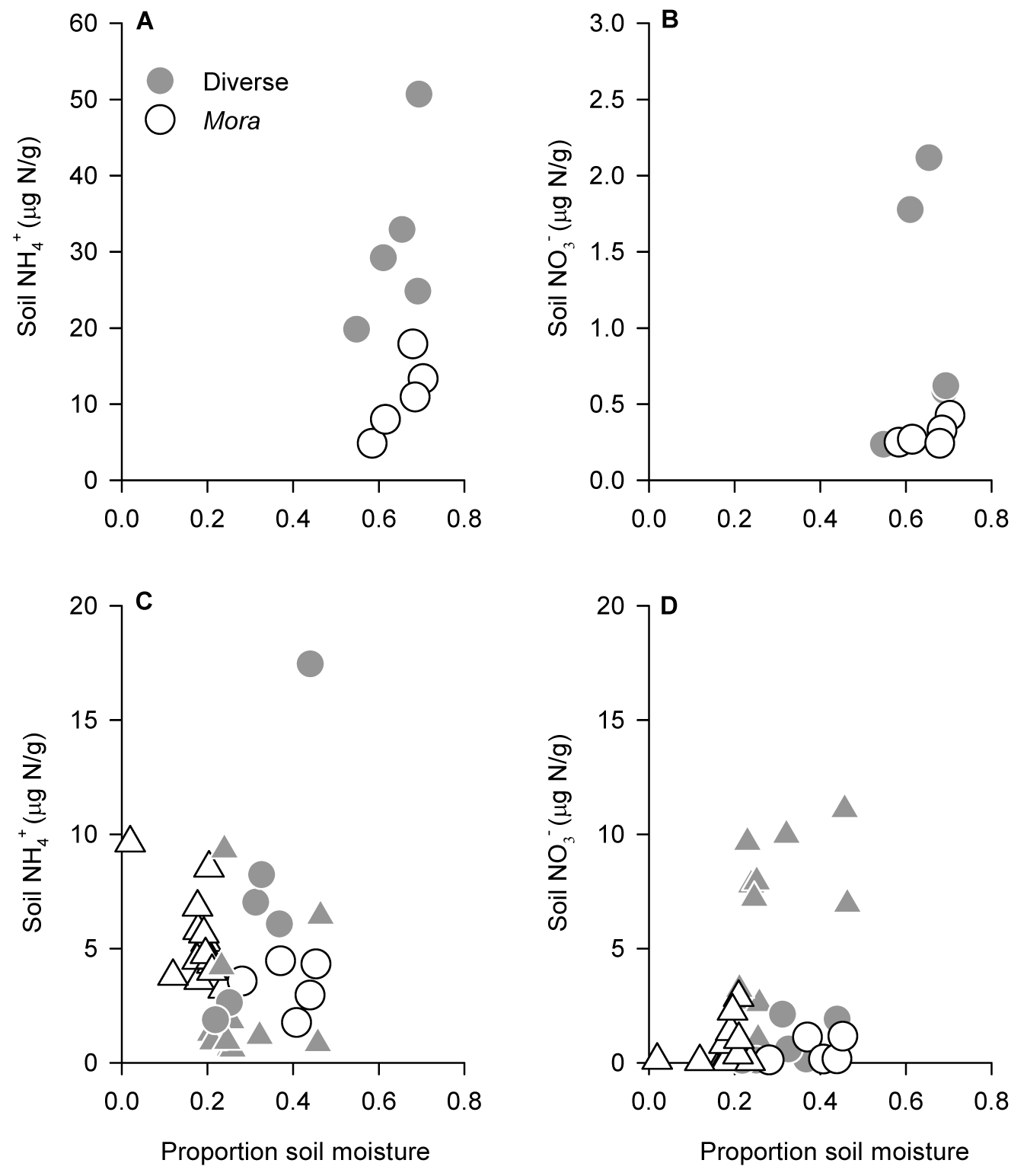

Supplement: Figure S1 — Concentrations of soil ammonium and nitrate as a function of gravimetric soil moisture. A, B) Concentrations of ammonium and nitrate in organic horizons of forest soils during the wet season (2012). C, D) Concentrations of ammonium and nitrate in mineral (0–15 cm) soils during dry (2010; triangles) and wet (2012; circles) seasons. Variation in soil moisture did not explain any significant (P>0.20) within- or between-forest variation in soil N. (TIF) [file pone.0070491.s001.tif]

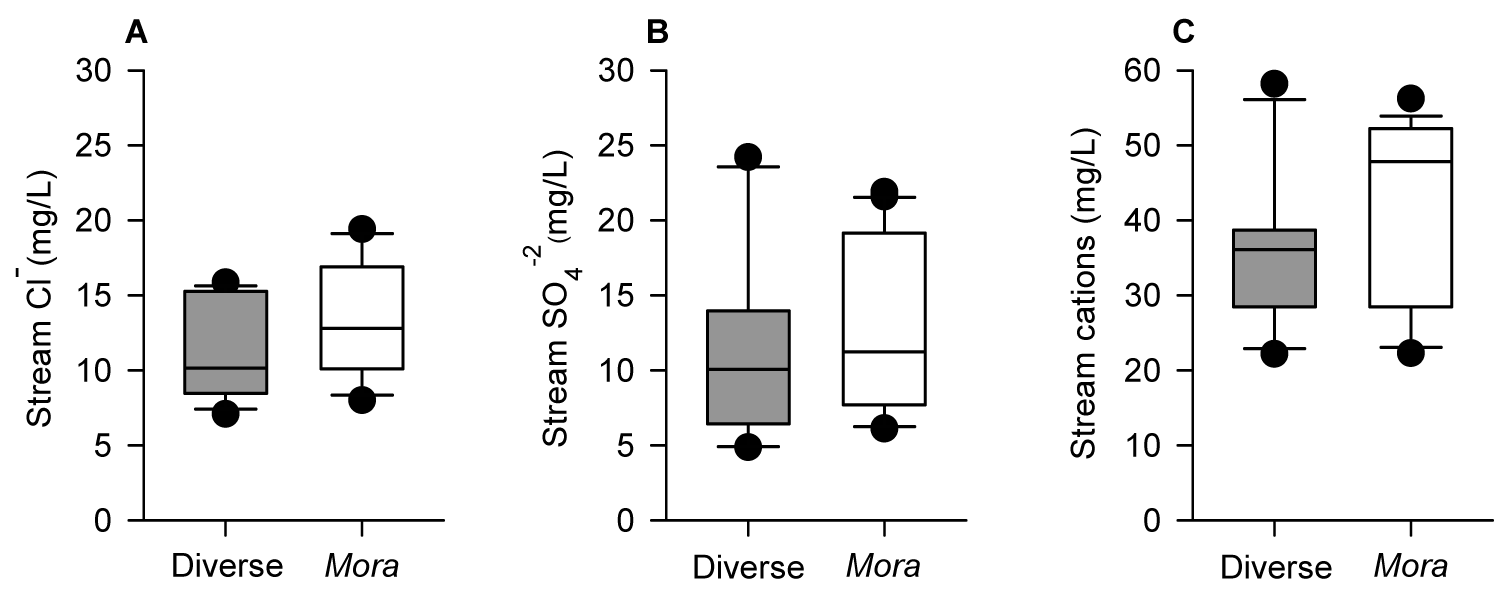

Supplement: Figure S2 — Concentrations of anions and cations in small watershed streams of diverse and Mora forests of Trinidad. (A) Box plots (median, 5th and 95th percentiles and outliers) of stream chloride in diverse (grey symbols n = 18) and Mora (white symbols, n = 15) forests. Stream chloride decreased strongly and synchronously between dry (2010) and wet (2011) years (P<0.001 for year effect) and differed between forest types (P = 0. 013 for type effect) with no significant year-type interaction (P = 0.139 for interaction term, df = 26, two-way ANOVA). Slightly higher chloride in Mora forest is opposite to that expected if dilution explained lower stream nitrate, is not consistent with other ions and water isotopes and could be explained by slightly closer proximity to the ocean. (B) Box plots (median, 5th and 95th percentiles) of stream sulphate in diverse (n = 18) and Mora (n = 15) forests. Sulphate also decreased significantly and synchronously from dry to wet seasons (P<0.001 for year effect) but did not differ between forest types across or within years (P = 0.098 for type effect, P = 0.611 for interaction term, df = 26, two-way ANOVA). (C) Box plots (median, 5th and 95th percentiles) of the sum of stream cations (K+, Na+, Ca++, Mg++). Cations showed significant and synchronous increases from 2009 to 2010 (P<0.001 for year effect) and no differences between forest types across or within years (P = 0.227 for type effect, P = 0.116 for interaction term, df = 29, two-way ANOVA). (TIF) [file pone.0070491.s002.tif]
